# Identification of *Francisella novicida* mutants that fail to induce prostaglandin E_2_ synthesis by infected macrophages

**DOI:** 10.3389/fmicb.2013.00016

**Published:** 2013-02-11

**Authors:** Matthew D. Woolard, Lydia M. Barrigan, James R. Fuller, Adam S. Buntzman, Joshua Bryan, Colin Manoil, Thomas H. Kawula, Jeffrey A. Frelinger

**Affiliations:** ^1^Department of Microbiology and Immunology, Louisiana State University Health Sciences Center at ShreveportShreveport, LA, USA; ^2^Department of Immunobiology, University of ArizonaTucson, AZ, USA; ^3^Department of Microbiology and Immunology, University of North Carolina at Chapel HillChapel Hill, NC, USA; ^4^DNA Identification Testing Division, Laboratory Corporation of AmericaBurlington, NC, USA; ^5^Department of Genome Sciences, University of WashingtonSeattle, WA, USA

**Keywords:** *Francisella*, prostaglandin E_2_

## Abstract

*Francisella tularensis* is the causative agent of tularemia. We have previously shown that infection with *F. tularensis* Live Vaccine Strain (LVS) induces macrophages to synthesize prostaglandin E_2_ (PGE_2_). Synthesis of PGE_2_ by *F. tularensis* infected macrophages results in decreased T cell proliferation *in vitro* and increased bacterial survival *in vivo*. Although we understand some of the biological consequences of *F. tularensis* induced PGE_2_ synthesis by macrophages, we do not understand the cellular pathways (neither host nor bacterial) that result in up-regulation of the PGE_2_ biosynthetic pathway in *F. tularensis* infected macrophages. We took a genetic approach to begin to understand the molecular mechanisms of bacterial induction of PGE_2_ synthesis from infected macrophages. To identify *F. tularensis* genes necessary for the induction of PGE_2_ in primary macrophages, we infected cells with individual mutants from the closely related strain *F. tularensis* subspecies *novicida* U112 (U112) two allele mutant library. Twenty genes were identified that when disrupted resulted in U112 mutant strains unable to induce the synthesis of PGE_2_ by infected macrophages. Fourteen of the genes identified are located within the *Francisella* pathogenicity island (FPI). Genes in the FPI are required for *F. tularensis* to escape from the phagosome and replicate in the cytosol, which might account for the failure of U112 with transposon insertions within the FPI to induce PGE_2_. This implies that U112 mutant strains that do not grow intracellularly would also not induce PGE_2_. We found that U112 *clpB*::Tn grows within macrophages yet fails to induce PGE_2_, while U112 *pdpA*::Tn does not grow yet does induce PGE_2_. We also found that U112 *iglC*::Tn neither grows nor induces PGE_2_. These findings indicate that there is dissociation between intracellular growth and the ability of *F. tularensis* to induce PGE_2_ synthesis. These mutants provide a critical entrée into the pathways used in the host for PGE_2_ induction.

## INTRODUCTION

*Francisella tularensis* is a facultative intracellular bacterium and the causative agent of tularemia. *F. tularensis* has a low infective dose, high morbidity, and can persist in the environment (Ellis et al., 2002). *F. tularensis* has also been produced as a bioweapon (Dennis et al., 2001), and is classified as a Category A Select Agent. There are four major subspecies of *F. tularensis*: *F. tularensis* subspecies *tularensis*, *F. tularensis* subspecies *holarctica*, *F. tularensis* subspecies *mediasiatica*, and *F. tularensis* subspecies *novicida*. *F. tularensis*, *F. holarctica* (including the live vaccine strain, LVS), and *F. novicida* all cause a fulminate disease in mice that is similar to tularemia in humans (Rick Lyons and Wu, 2007). There are clear differences in virulence between strains in mice. *F. novicida*, *F. holarctica*, and *F. tularensis* can have an LD_50_ of less than 10 organisms in intranasally inoculated mice, while *F. holarctica* LVS LD_50_ in mice is much higher (Pechous et al., 2009). Each strain varies in its capacity to cause disease in humans. *F. novicida* is highly attenuated in humans, only causing disease in immuno-compromised individuals (Hollis et al., 1989; Hand et al., 2012). *F. holarctica* is highly infectious in humans, but causes a milder form of tularemia compared to *F. tularensis*. *F. holarctica* LVS is highly attenuated for disease in humans but can cause disease in immunocompetent individuals (Tigertt, 1962; Hornick and Eigelsbach, 1966; Ellis et al., 2002). Though each strain has a different level of virulence in humans, they share high nucleotide sequence identity. *F. novicida* shares 95% nucleotide sequence identity with *F. tularensis* and *F. holarctica* (Rohmer et al., 2007), suggesting that homologous proteins function via similar mechanisms.

Key to *F. tularensis*’ virulence is its ability to escape the phagosome and replicate within the cytosol of host cells. Previous studies have identified over 200 genes that are necessary for intracellular growth of *F. tularensis* (Qin and Mann, 2006; Weiss et al., 2007; Kraemer et al., 2009; Asare and Abu Kwaik, 2010; Asare et al., 2010). Some of the genes required for escape from the phagosome and intracellular growth reside within the *Francisella* pathogenicity island (FPI; Barker et al., 2009). The FPI is a set of 16 genes that are highly conserved among all subspecies of *F. tularensis* (Barker et al., 2009). The FPI likely encodes a secretion system that is related to the recently discovered type VI secretion systems (T6SS; Nano and Schmerk, 2007; Ludu et al., 2008). The T6SS is involved in the virulence of several bacterial pathogens (Mougous et al., 2006; Pukatzki et al., 2006; Shalom et al., 2007; Ma and Mekalanos, 2010). Several regulators of FPI expression have been described. Two of the best studied are MglA and SspA, which positively regulate the transcription of FPI genes (Baron and Nano, 1998; Lauriano et al., 2004; Charity et al., 2007). The mechanisms by which FPI proteins promote *F. tularensis* escape and intra-macrophage growth are unknown. There is evidence that translocated products of T6SS in other bacteria are capable of modulating host immune responses (Pukatzki et al., 2007; Ma and Mekalanos, 2010; Suarez et al., 2010a,b). Though FPI gene products are clearly involved in phagosome escape and intracellular growth, the ability of these gene products to induce immunomodulatory responses has not been demonstrated to date.

Prostaglandin E_2_ (PGE_2_) synthesis induced by LVS from host cells alters both innate and adaptive immune responses. We demonstrated that *F. tularensis* LVS was capable of inducing macrophages to synthesize PGE_2_ and that this was independent of intracellular growth of *F. tularensis* (Woolard et al., 2007). *In vitro*, LVS-induced PGE_2_ synthesis inhibits T cell proliferation and skews their phenotypic development from IFN-γ^+^ T cells to IL-4^+^ T cells (Woolard et al., 2007). Through an indirect mechanism, PGE_2_ induces ubiquitin-mediated degradation of MHC II which results in decreased MHC II protein levels on the surface of macrophages (Wilson et al., 2009). Decreased MHC II surface expression would decrease the antigenic stimulatory capacity of these macrophages, likely making them less capable of activating *F. tularensis*-specific T cells. T cells are required for both clearance of *F. tularensis* and generation of long-term immune protection (Yee et al., 1996), thus the biological activity of PGE_2_ would be beneficial to *F. tularensis* survival *in vivo*. LVS-induced PGE_2_ synthesis during respiratory tularemia inhibits the generation of beneficial T cell response. The inhibition of PGE_2_ synthesis *in vivo* by indomethacin leads to increased number of IFN-γ^+^ T cells and decreased bacterial burden (Woolard et al., 2008). It is clear that induction of PGE_2_ synthesis is an important immune modulation mechanism utilized by *F. holarctica* to persist in the host.

Presently, none of the *F. tularensis* product(s) responsible for the induction of PGE_2_ synthesis in eukaryotic cells are known. Several bacterial products have been identified that are capable of inducing PGE_2_ synthesis. Bacterial peptidoglycan, LPS, and CpG DNA can up-regulate prostaglandin synthesis through interactions with TLR2, TLR4, and TLR9, respectively (Chen et al., 2001, 2004; Smith et al., 2002; Uematsu et al., 2002; Treffkorn et al., 2004). It is not known if *F. tularensis* is capable of inducing PGE_2_ through a similar mechanism. To date, few *F. tularensis* TLR ligands have been identified. *F. tularensis* LpnA and FTT1103 have been reported to be TLR2 ligands and DnaK a TLR4 ligand (Ashtekar et al., 2008; Forestal et al., 2008; Thakran et al., 2008). *F. tularensis* LPS fails to or only weakly stimulates a cytokine response by host cells (Kieffer et al., 2003; Hajjar et al., 2006). If *F. tularensis* LPS does stimulate host cells, it is likely in a TLR4 independent manner. Both TLR2 and TLR4 deficient macrophages produce PGE_2_ after infection (Woolard et al., unpublished data).

In this study we demonstrate that along with LVS, *F. novicida* U112 (U112), and *F. tularensis* subspecies *tularensis* Schu S4 (Schu S4) induce PGE_2_ synthesis by macrophages. We tested a *F. novicida* (U112) comprehensive transposon mutant library to identify genes necessary for induction of PGE_2_ synthesis by infected bone marrow-derived macrophages (BMDMs). This library allowed us to identify 20 genes that when disrupted result in U112 strains that are unable to induce the synthesis of PGE_2_ by infected macrophages. Identified genes included genes of the FPI and regulators of the FPI. All genes identified are highly conserved among all sequenced strains of *F. tularensis* (Charity et al., 2007, 2009; Nano and Schmerk, 2007; Meibom et al., 2008). We also demonstrate that the ability of *F. novicida* to induce PGE_2_ synthesis is likely not dependent on phagosome escape nor intracellular growth. This work likely suggests that the FPI is involved in immune modulation along with previously established mechanisms of phagosomal escape and intracellular growth.

## MATERIALS AND METHODS

### BACTERIA AND MOUSE STRAINS

The *F. tularensis* subspecies *holarctica* LVS was obtained from ATCC (29684; American Type Culture Collection, Manassas, VA, USA; Cowley and Elkins, 2003), the *F. tularensis* subspecies* novicida* U112 strain was previously published (Larson et al., 1955), and the *F. tularensis* subspecies *tularensis* Schu S4 strain (catalog no. NR-643) was obtained from the Biodefense Emerging Infections Research Resources Repository (Manassas, VA, USA). The two allele transposon library was previously described (Gallagher et al., 2007). For all studies, except for the original screen, *F. novicida* was propagated on tryptic soy agar supplemented with 0.1% cysteine while the *F. novicida* transposon mutants were propagated on the same agar with the addition of 20 μg/ml of kanamycin. *F. holarctica* LVS and *F. tularensis* Schu S4 were propagated on chocolate agar. Inocula were generated by collecting plate grown bacteria and diluting them in PBS to reach an OD_600_ of 1.00. Inocula were then diluted into appropriate cell culture medium for inoculation.

The *F. novicida* two allele transposon library was previously described (Gallagher et al., 2007). The LVS ∆*mglA*, LVS ∆*sspA*, LVS *∆mglA pmglA*, and LVS ∆*sspA psspA* were previously published (Fuller et al., 2009). The *dotU* deletion construct was made by splice overlap extension PCR retaining the start and stop codons of *dotU* and fusing the first four and last two codons in frame and retaining 0.8 kb of flanking sequence. The constructs were cloned into the suicide vector pMP590 and sequenced to confirm the integrity of the DNA sequence. The LVS *dotU* mutant was generated by allelic exchange, selection for plasmid co-integrates, and counter selection on sucrose containing media to identify plasmid and *dotU* allele resolution as described (Fuller et al., 2008). The following primers were utilized to generated the SOE fragment; FTL0119 5′ ext 5′-GAGTTTTTTCCACCTCTGAGGATGTTTC, FTL0119 5′ int 5′-GAAAGACTTTAAAGAGATAGAATAATAAGGGTAAGAGGAGATTTATATGAGTCAGATAATATC, FTL0119 3′ int 5′-CTCCTCTTACCCTTATTATTCTATCTCTTTAAAGTCTTTCATTTATAATATCCTTTATATAGAG, FTL0119 3′ ext 5′-CATACATATTTAACCAAGTATTAGAAGATAATGGCTCAG. Loss of the wild-type and retention of the deletion *dotU* alleles were confirmed by PCR. Since *dotU* is duplicated in the LVS genome, a second round of mutagenesis was performed on the single *dotU* mutant strain to create an LVS *dotU* double-deletion strain. Plasmids for complementation were created by ligating cloned region of *dotU* into the PKK MCS plasmid. *dotU* expression from the PKK MCS plasmid was regulated by the putative PI promoter. The following primers were used; FTL0119 forward 5′-CTTAATTAAATGAAAGACTTTAAAGAGATAGAAATTATTCTAGATATTATAAAAAC, FTL0119 reverse 5′-TGTCGACCCAGCTTAATAAAATTAGTAAGCTTAAAAGAAACAGTC.

C57Bl/6J (B6) mice were purchased from the Jackson Laboratory (Bar Harbor, ME). All animals used in this study were maintained under specific pathogen-free conditions in the American Association of Laboratory Animal Care-accredited University of North Carolina Department of Laboratory Animal Medicine Facilities or American Association of Laboratory Animal Care-accredited Louisiana State University Health Science Center at Shreveport Animal Medicine Facilities. All work was approved by each facility’s Animal Care and Use Committee (UNC #04-200, LSUHSC P10-010).

### GENERATION OF BONE MARROW-DERIVED MACROPHAGES

Bone marrow cells from B6 mouse femurs were cultured in 30% L cell-conditioned medium as previously described (Woolard et al., 2007). Briefly bone marrow cells were flushed from B6 mouse femurs and incubated for 7 days on non-tissue culture-treated 15-cm^2^ dishes with L cell-conditioned medium as a source of GM-CSF. Following differentiation, non-adherent cells were removed by multiple washes with PBS and BMDMs were removed from plates by incubation with 10 mM EDTA in PBS. Since L cell-condition media and FBS batches can affect the amount of PGE_2_ induction by infected macrophages we utilized the same L cell-conditioned media and FBS batches for each series of experiments to minimize variability in PGE_2_ synthesis between experiments.

### BONE MARROW-DERIVED MACROPHAGE INFECTIONS

Bone marrow-derived macrophages were plated in 96-well flat bottom plates (10^5^/well). Macrophages were allowed to adhere for 2 h. Macrophages were mock infected or infected with LVS, Schu S4, U112, or U112 transposon insertion strains at different multiplicity of infections (MOIs) as indicated. Bacteria were centrifuged onto the macrophage monolayer at 300 *g* for 5 min to allow closer contact and more efficient infection. Two hours after inoculation, extracellular bacteria were killed by the addition of 50 μg/ml of gentamicin for 45 min. Supernatants were removed and cells were washed with antibiotic-free complete medium. Fresh antibiotic-free complete medium was added and cells were incubated for 24 h. Supernatants were then collected and spun at 300 *g* for 10 min to remove eukaryotic cells. Supernatants were sterilized by UV. Representative supernatants were plated onto chocolate agar after UV treatment to ensure complete killing of *F. tularensis*. Supernatant was then stored at -80°C until needed.

### IDENTIFICATION OF TRANSPOSON INSERTION STRAINS

The transposon library has previously been described (Gallagher et al., 2007). In brief, the 3,050-member library includes two insertion alleles in 1488 genes (the majority of total *Francisella* ORFs). The alleles chosen were primarily insertions positioned between 5 and 70% within the ORF and are thus likely to represent null mutations. After single-colony purification, the mutants were arrayed in 96-well format and sequence-mapped to confirm their identities (see Table 2 of Gallagher et al., 2007 for the summary of this information).

The two allele mutant library was screened in a 96-well format. Transposon insertion strains were grown up in 96-well deep well plates containing 1 ml of Tryptic Soy broth containing 15 μg/ml carbenicillin, 20 μg/ml kanamycin, and supplemented with 0.1% l-cysteine-HCl. After over-night growth an aliquot of supernatants from each transposon insertion strain was taken and OD_600_ was determined. MOI were normalized by average plate OD_600_. B6 BMDM was inoculated at an MOI of 500:1 to guarantee sufficient inocula in each well to induce PGE_2_ synthesis. In our experience increasing MOI inocula increases the number of macrophages infected. Twenty-four hours after inoculation supernatants were collected and then stored at -80°C until needed.

### PGE_2_ ASSAY

Prostaglandin E_2_ in cell culture supernatants was measured using a commercial PGE_2_ enzyme immunoassay kit (Assay Design, Ann Arbor, MI, USA) as per manufacturer’s instructions. Transposon insertion strains were deemed defective in the ability to induce PGE_2_ when the levels of PGE_2_ by any transposon insertion strain were three standard deviations (SD) below the mean of the entire plate.

### BACTERIAL GROWTH ASSAY

Macrophages were mock infected or infected with U112, U112 *clpB*::Tn, U112 *pdpA*::Tn, or U112 *iglC*::Tn strains at an MOI of 100:1. At 4 and 24 h post-inoculation, supernatants were removed. 100 μl of 0.05% sodium dodecyl sulfate in PBS was used to lyse the BMDM. Samples were transferred to tubes containing 900 μl PBS and vortexed on high setting for 1 min. Samples were serially diluted and plated on chocolate agar to determine bacterial numbers.

### CONFOCAL AND TRANSMISSION ELECTRON MICROSCOPY

J774.1 macrophages (from ATCC #TIB-67) were seeded on coverslips at a density of 6 × 10^5^ cells/well. Prior to infection, bacteria were carboxyfluorescein succinimidyl ester (CFSE) labeled as previously described (Bosio and Dow, 2005) with the following modifications: CFSE was added to bacteria at a final concentration of 5 μM and incubated for 20 min at 37°C. Macrophages were inoculated with CFSE-labeled U112, U112 *clpB*::Tn, U112 *pdpA*::Tn, or U112 *iglC*::Tn mutants at an MOI of 200:1. Bacteria were centrifuged onto the macrophages at 300 *g* for 5 min. Two hours after inoculation, extracellular bacteria were killed by the addition of 25 μg/ml of gentamicin for 45 min and then media was replaced with antibiotic-free media. At 4 h post-inoculation, LAMP-1 association with bacteria was determined as previously described (Schmerk et al., 2009a). Briefly, wells were washed with PBS and fixed for 20 min at room temperature with 2% (w/v) formaldehyde and 1% (w/v) sucrose in PBS. Cells were permeabilized using methanol. Coverslips were blocked with 5% bovine serum albumin, incubated overnight at 4°C with anti-mouse LAMP-1 (1D4B eBioscience), washed three times with PBS, and stained with donkey anti-rat IgG Alexafluor594 secondary antibody (Invitrogen) for 2 h at room temperature. After three PBS washes, the coverslips were mounted in DAPI-containing mounting media (Vector Laboratories, Inc.) to label the DNA. Cells were imaged using a Leica SP2 Laser Scanning Confocal Microscope using a 63× oil immersion lens. A minimum of 20 cells per strain were captured. To remove subjectivity in determining co-localization of bacteria with LAMP-1 images were analyzed using Volocity software (Improvision/Perkin Elmer) to determine bacterial association with LAMP-1. Co-localization was determined by the shared of red and green pixels at the same location. To determine whether a bacterium resided in a LAMP-1 positive vesicle, the voxel spy tool was used to closely examine whether the LAMP-1 red pixels surrounded the CFSE green pixels that labeled the bacterium. If the red pixels surrounded >50% of the green pixels, the bacterium was categorized as residing within a LAMP-1+ vesicle.

B6 BMDMs were inoculated with U112, U112 *clpB*::Tn, U112 *iglC*::Tn, or U112 *pdpA*::Tn at an MOI of 500:1 to maximize the number of infected BMDMs. 2 h after inoculation, the media was removed and replaced with media containing 50 μg/ml gentamicin (Sigma-Aldrich, St. Louis, MO, USA). Gentamicin-containing media was removed 1 h after treatment and replaced with antibiotic-free media. Four hours post-inoculation, the BMDM monolayer was fixed using gluteraldehyde and post-fixed with osmium tetroxide. Images were obtained using a Phillips CM-12 transmission electron microscope using 25,000× magnification.

### STATISTICAL ANALYSIS

Student’s *t*-tests were used for statistical analysis between two group experiments. Multi group comparisons were done by ANOVA followed by Dunnett’s Multiple Comparison Test. When appropriate, data were logarithmically transformed before statistical analysis and confirmed by a demonstrated increase in power of the test after transformation of the data. Data analysis on the rescreen (**Figure 2**) was accomplished by one-way ANOVA analysis followed by Student’s *t*-test. A *p*-value ≤ 0.05 was considered statistically significant.

## RESULTS

### *F. tularensis* SUBSPECIES *novicida* AND *tularensis* INDUCED THE SYNTHESIS OF PGE_2_ BY INFECTED MACROPHAGES

**FIGURE 1:**
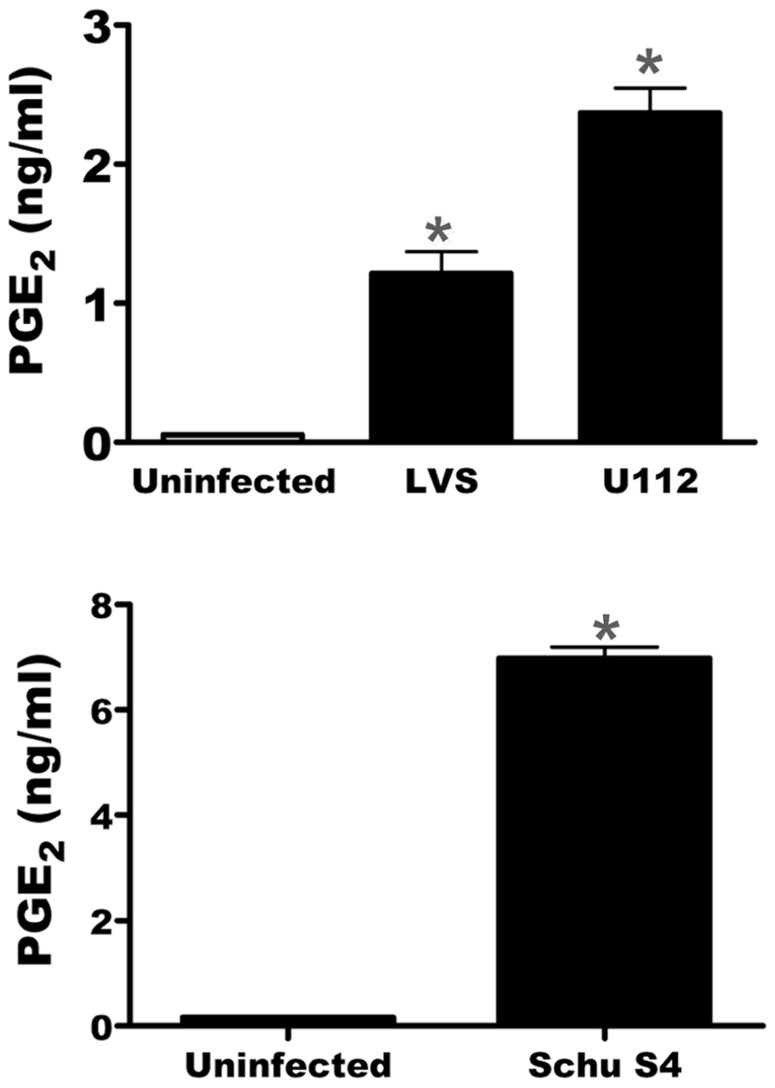
**U112 and Schu S4 induces the synthesis of PGE_2_ from bone marrow-derived macrophages (BMDMs)**. BMDMs were either mock inoculated or inoculated with LVS, U112, or Schu S4 at an MOI of 200:1. Twenty-four hours after inoculation supernatants were collected and PGE_2_ concentration was determined. Data represents three independent experiments and expressed as the mean ± SEM. Asterisk “*”denotes statistical difference (*p* ≤ 0.05) from uninfected BMDM (*n* = 3).

We have previously demonstrated that *F. tularensis* subspecies *holarctica* LVS induces PGE_2_ synthesis in infected macrophages. To enable the use of the two allele transposon mutant library we needed to determine if the ability to induce PGE_2_ synthesis by infected macrophages is shared among *Francisella* subspecies. We tested both *F. novicida* U112 and *F. tularensis* Schu S4 for their ability to induce B6 BMDMs to synthesize PGE_2_ upon infection. We inoculated BMDM with LVS, U112, or Schu S4 at an MOI of 200:1. All strains tested were capable of inducing synthesis of PGE_2_ by infected macrophages (**Figure 1**). This demonstrates that the ability to induce PGE_2_ synthesis is conserved among *F. tularensis* strains.

### SCREENING THE TWO ALLELE MUTANT LIBRARY IDENTIFIES SEVERAL GENES NECESSARY FOR THE *Francisella* INDUCTION OF PGE_2_ BY INFECTED MACROPHAGES

**FIGURE 2:**
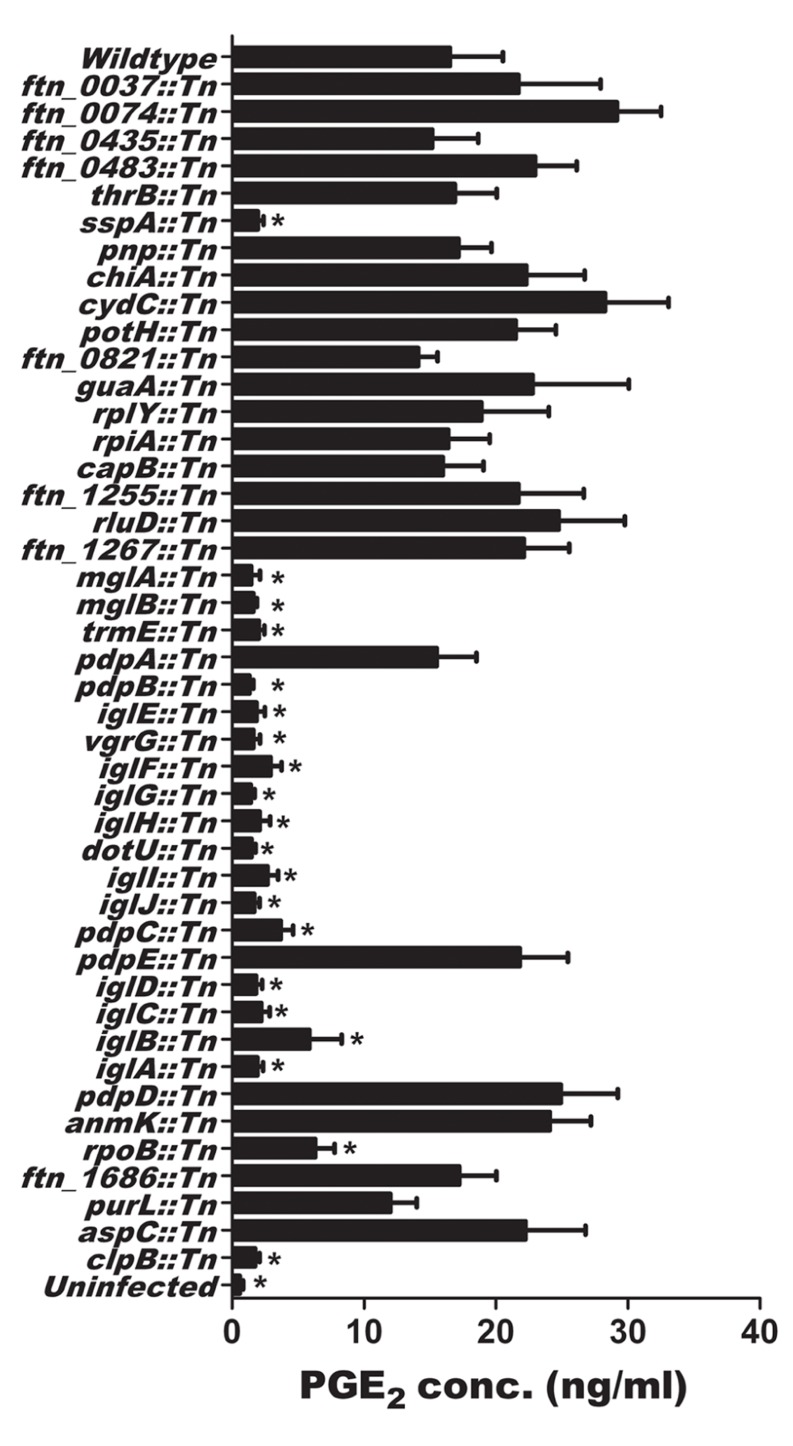
**Identification of U112 genes necessary for the induction of PGE0_2_ from bone marrow-derived macrophages (BMDMs)**. BMDMs were inoculated with *F. novicida* U112 or individual transposon insertion strains at an MOI of 200:1. Twenty-four hours after inoculation, supernatants were collected and PGE_2_ concentration was determined. Each transposon insertion mutant strain was tested four times. Bars represents the mean of all independent transposon insertions mutants within the same gene ± SEM, Asterisk “*” denotes statistical difference (*p* ≤ 0.05) from U112 inoculated BMDM (*n* ≥ 4).

Since we demonstrated that U112 induced macrophage synthesis of PGE_2_, we used the *F. novicida* two allele transposon mutant library (Gallagher et al., 2007) to identify mutants that were unable to induce PGE_2_ synthesis. During the initial testing of the 3050 *F. novicida* U112 transposon mutants, we defined a *F. novicida* U112 transposon mutant as defective in induction of PGE_2_ synthesis by infected BMDM when BMDM produced relative PGE_2_ amounts that were three SD lower than the plate average amount of PGE_2_. The use of the three SD rule allowed us to minimize the likelihood (0.3%) of identifying false positives. The initial screen identified 33 genes that when disrupted made *F. novicida* unable to induce PGE_2_ synthesis by infected macrophages (Table S1 in Supplementary Material). This included 10 genes located in the FPI. We retested all U112 transposon insertion mutants with transposon insertions in the identified 33 genes. Furthermore, since the initial screen identified 10 genes of the FPI, we included all FPI transposon mutants within the two allele transposon mutant library in this rescreen to ensure these genes important in pathogenesis were carefully evaluated. BMDMs were inoculated with individual transposon insertion strains (89 mutants representing the original 33 genes identified and 10 genes from the FPI not originally identified) at an MOI of 200:1 and PGE_2_ levels were measured 24 h post-inoculation (**Figure 2**). We utilized an MOI of 200:1 since we have previously demonstrated this MOI results in a reproducible significant increase in detectable PGE_2_ from infected macrophages (Woolard et al., 2007). Each U112 transposon mutant was tested a minimum of four times. No difference was noted between strains with insertions in the same gene; as such the values were combined for representation in **Figure 2**. We were able to confirm 20 genes that when disrupted resulted in *F. novicida* strains that did not induce the synthesis of PGE_2_ by infected BMDM (**Figure 2**). With the exception of *mglA* and *rpoB*, which were only represented once, each gene identified encodes a product involved in the induction of PGE_2_ that was represented at least twice in the U112 two allele transposon mutant library. The genes identified in the screen of the two allele mutant library are summarized in **Table 1**. The identified genes were located in the FPI or were genes that encode some of the previously identified regulators of the FPI (*sspA*,* mglA*, *mglB*, and *trmE*) with the exception of *rpoB* and *clpB* (Baron and Nano, 1998; Charity et al., 2007, 2009; Guina et al., 2007; Nano and Schmerk, 2007). These genes are highly conserved in all *F. tularensis* subspecies sequenced to date (Charity et al., 2007, 2009; Nano and Schmerk, 2007; Meibom et al., 2008). Of note, not all genes encoded within the FPI are necessary for U112-induced PGE_2_ synthesis as *pdpA::Tn*, *pdpD::Tn*, and *pdpE::Tn* were able to induce PGE_2_ synthesis similarly to wild-type U112. Thus, the screen identified 20 *F. novicida* genes that are necessary for the induction of PGE_2_.

**Table 1:** Genes required for *Francisella* induction of PGE2 synthesis in *Francisella*-infected macrophages.

ORF	Gene	Function (postulated)
FTN_0549	sspA	Regulate virulence genes
FTN_1290	mglA	Regulate virulence genes
FTN_1291	mglB	Regulate virulence genes
FTN_1298	trmE	tRNA modification, GTPase activity
FTN_1310	pdpB	Unknown
FTN_1311	iglE	Unknown
FTN_1312	vgrG	Secreted
FTN_1313	iglF	Unknown
FTN_1314	iglG	Unknown
FTN_1315	iglH	Unknown
FTN_1316	dotU	Unknown
FTN_1317	iglI	Unknown
FTN_1318	iglJ	Unknown
FTN_1319	pdpC	Unknown
FTN_1321	iglD	Replication in cytosol
FTN_1322	iglC	Escape from phagosome
FTN_1323	iglB	Unknown
FTN_1324	iglA	Unknown
FTN_1568	rpoB	DNA directed RNA polymerase subunit beta
FTN_1743	clpB	Chaperone, ATPase activity

### *F. tularensis* LVS MUTANT STRAINS WITH DELETIONS OF *mglA*, *sspA*, OR *dotU* DO NOT INDUCE PGE_2_ SYNTHESIS FROM INFECTED MACROPHAGES

**FIGURE 3:**
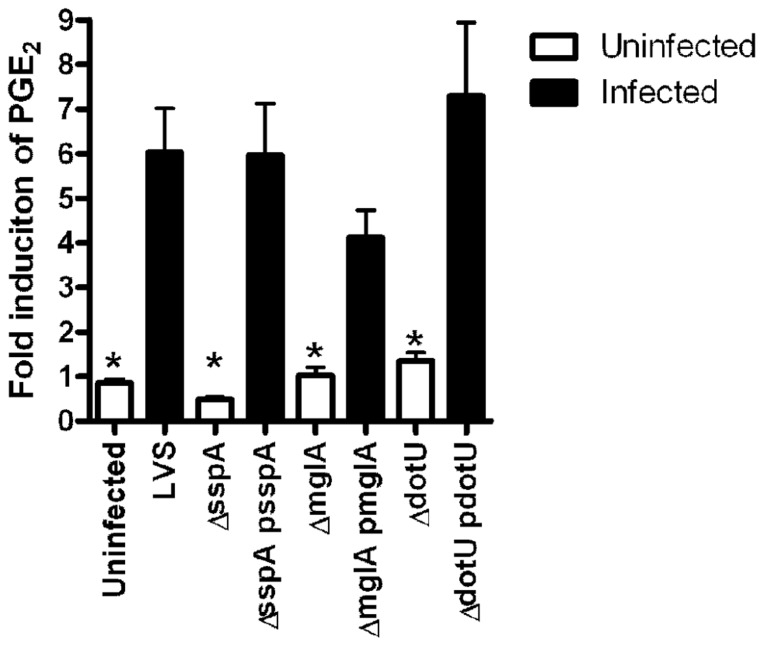
***mglA*, *sspA*, and *dotU* are necessary for LVS induction of PGE_2_ synthesis**. Bone marrow-derived macrophages were inoculated with LVS, LVS∆*mglA*, LVS∆*mglA* (*pmglA*), LVS∆*sspA*, LVS∆*sspA* (*psspA*), LVS∆*dotU*, LVS∆*dotU* (*pdotU*), or LVS at an MOI of 200:1. Twenty-four hours after inoculation the levels of PGE_2_ were determined. Experiments were done in triplicate; error bars represent SEM (*n* = 3).

To begin to address if the genes identified in U112 also encode products that contribute to LVS to induce PGE_2_ synthesis by infected macrophages we utilized clean deletion mutants. Two of the genes identified in the screen of the two allele library, U112 *mglA*::Tn and U112 *sspA*::Tn, encode positive transcriptional regulators (Baron and Nano, 1998; Lauriano et al., 2004; Charity et al., 2007). We also identified several genes of the FPI, including *dotU*. DotU is necessary for stabilization of the FPI secretion apparatus, and mutants lacking *dotU* do not have a functional FPI secretion system (Broms et al., 2012). To examine the possibility that LVS mutants lacking *mglA*, *sspA*, or *dotU* do not to induce PGE_2_ synthesis, we tested these mutant strains for induction of PGE_2_ synthesis by BMDMs. BMDMs were inoculated with LVS, LVS∆*mglA*, LVS∆*mglA* (*pmglA*), LVS∆*sspA*, LVS∆*sspA* (*psspA*), LVS∆*dotU*, or LVS∆*dotU* (*pdotU*) at an MOI of 200:1. Twenty-four hours after inoculation the levels of PGE_2_ were determined. Neither LVS∆*mglA*, LVS∆*sspA* nor LVS∆*dotU* mutant strains induce significant PGE_2_ synthesis from infected macrophages (**Figure 3**). This phenotype was reversed by *trans* complementation with the appropriate plasmid (**Figure 3**), suggesting that U112 and LVS induce PGE_2_ synthesis through similar mechanisms.

### DISSOCIATION OF INTRACELLULAR GROWTH AND INDUCTION OF PGE_2_ BY *Francisella*

Escape from the phagosome and replication in the cytosol of host cells are critical for *F. tularensis* survival. All of the genes identified in this screen have been identified in other screens examining disease pathogenesis and intracellular growth (Maier et al., 2007; Su et al., 2007; Weiss et al., 2007; Kraemer et al., 2009; Asare and Abu Kwaik, 2010; Asare et al., 2010). Thus, it may be that failure to either escape the phagosome or replicate explains why these *F. novicida* mutants did not to induce PGE_2_ synthesis. Previous studies that examined infection of macrophages by *F. novicida* *pdpA*::Tn and ∆*pdpA* strains demonstrated that PdpA is required for escape from the phagosome (Schmerk et al., 2009a,b). Similarly, IglC has been shown to be required for *F. novicida* and *F. holarctica* phagosomal escape (Lindgren et al., 2004; Bonquist et al., 2008). In contrast, *F. holarctica* mutants with a transposon insertion in *clpB* escape the phagosome and replicate (Meibom et al., 2008). The characterization of the trafficking phenotypes of *Francisella* strains with mutations in *clpB*, *pdpA*, and *iglC* suggested we could use the two allele mutant library *clpB::*Tn, *pdpA::*Tn, and *iglC::*Tn mutant strains as tools to investigate the requirement of escape and intracellular growth for PGE_2_ induction. We understand these experiments do not prove that these genes are necessarily involved in the induction of PGE_2_, but rather eliminate or confirm if either the act of escaping the phagosome or replicating in the cytosol is what is necessary and sufficient to induce PGE_2_ synthesis in macrophages.

**FIGURE 4:**
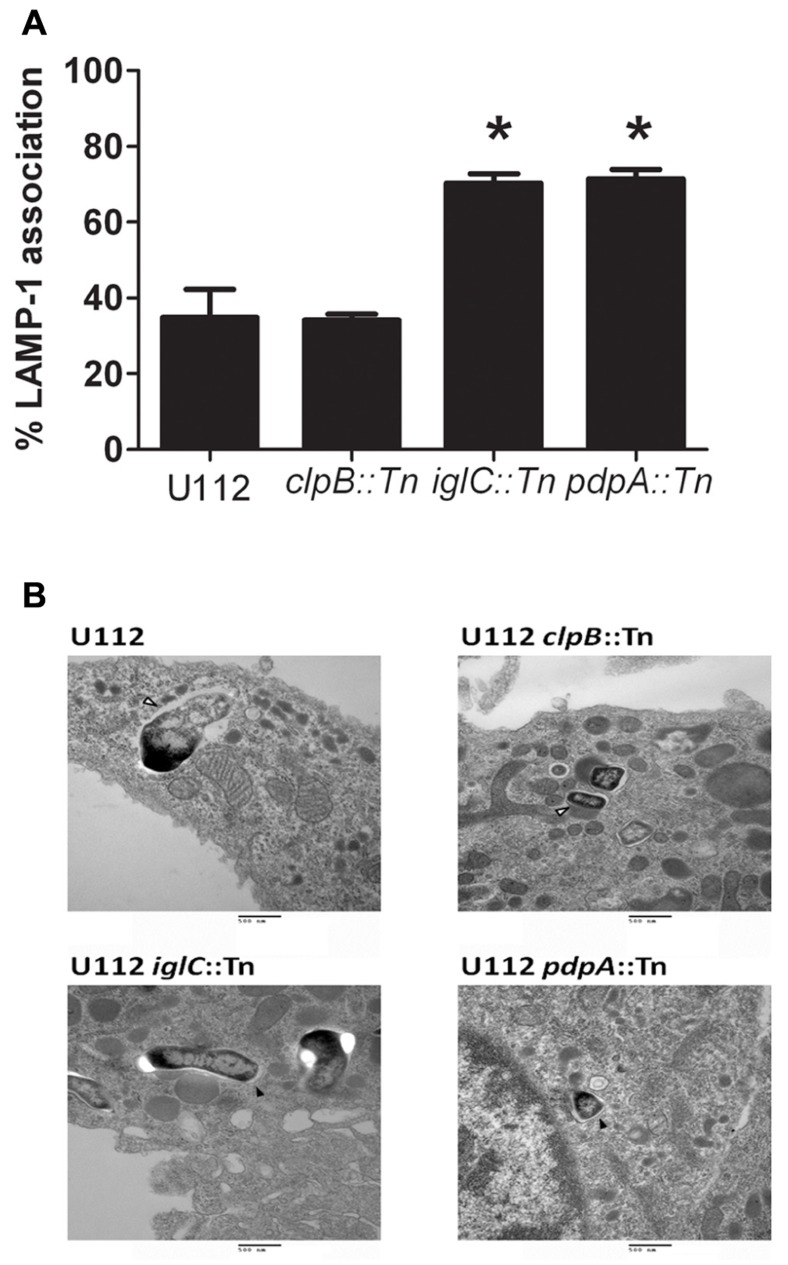
**Induction of PGE_2_ does not require full escape from the phagosome**. **(A)** Bacterial association with LAMP-1 was scored using Volocity (*n* ≥ 20 imaged cells per strain with an average of one bacterium per macrophage). Co-localization was determined by the shared of red and green pixels at the same location. Data represents three independent experiments and expressed as the mean ± SEM. Asterisk “*” denotes statistical difference (*p* ≤ 0.05) from U112-infected cells. **(B)** BMDMs were inoculated with U112, U112 *clpB*::Tn, U112 *iglC*::Tn, or U112 *pdpA*::Tn at an MOI of 500:1. Association of the bacterium with the phagosomal membrane was determined 4 h post-inoculation using transmission electron microscopy. Open arrowheads denote bacteria no longer surrounded by an intact phagosomal membrane. Filled arrowheads denote bacteria surrounded by a phagosomal membrane.

To determine the intracellular localization of these strains, we inoculated the J774.1 macrophage cell line, as we and others have successfully used this cell line in the past to examine intracellular localization of *F. tularensis* (Tempel et al., 2006; Fuller et al., 2008), at an MOI of 500:1 with CFSE labeled U112, U112 *clpB*::Tn, U112 *iglC*::Tn, and U112 *pdpA*::Tn and examined their association with LAMP-1 using Confocal microscopy (**Figure 4**). A high MOI was used to ensure our ability to identify intracellular bacteria and their respective intracellular localization. We confirmed that U112 infected J774 cells synthesize increased amounts of PGE_2_ upon both U112 and LVS infection compared to uninfected samples (data not shown)_._ We analyzed the associations of bacteria and LAMP-1 using Volocity image software and showed the percentage of bacteria associated with LAMP-1 by pixel association (**Figure 4A**). We found only 34% of U112 remained associated with LAMP-1 4 h post-inoculation. Similarly, 34% of U112 *clpB*::Tn remained associated with LAMP-1 4 h post-inoculation. In contrast, U112 *iglC*::Tn and U112 *pdpA*::Tn resided mainly in the phagosome 4 h post-inoculation displaying 71% and 70% LAMP-1 association, respectively. We confirmed the intracellular localization of U112 *clpB*::Tn, U112 *iglC*::Tn, and U112 *pdpA*::Tn by transmission electron microscopy (**Figure 4B**). These data indicate U112 *clpB*::Tn, U112 *iglC*::Tn, and U112 *pdpA*::Tn have intracellular trafficking patterns that are similar to those of previously published *clpB*, *iglC*, and *pdpA* transposon insertion strains (Lindgren et al., 2004; Bonquist et al., 2008; Meibom et al., 2008; Schmerk et al., 2009a,b). As noted above, these data show that the U112 *pdpA*::Tn mutant is able to induce PGE_2_ even though it was diminished in its ability to escape the phagosome. If PGE_2_ synthesis induction required phagosomal escape, we would expect U112 *pdpA*::Tn would not induce PGE_2_ synthesis, as seen with U112 *iglC*::Tn. However, *pdpA*::Tn does induce PGE_2_ at similar levels to wild-type U112 (**Figure 2**). Thus, our data suggest PGE_2_ induction is unaffected by intracellular trafficking/localization.

**FIGURE 5:**
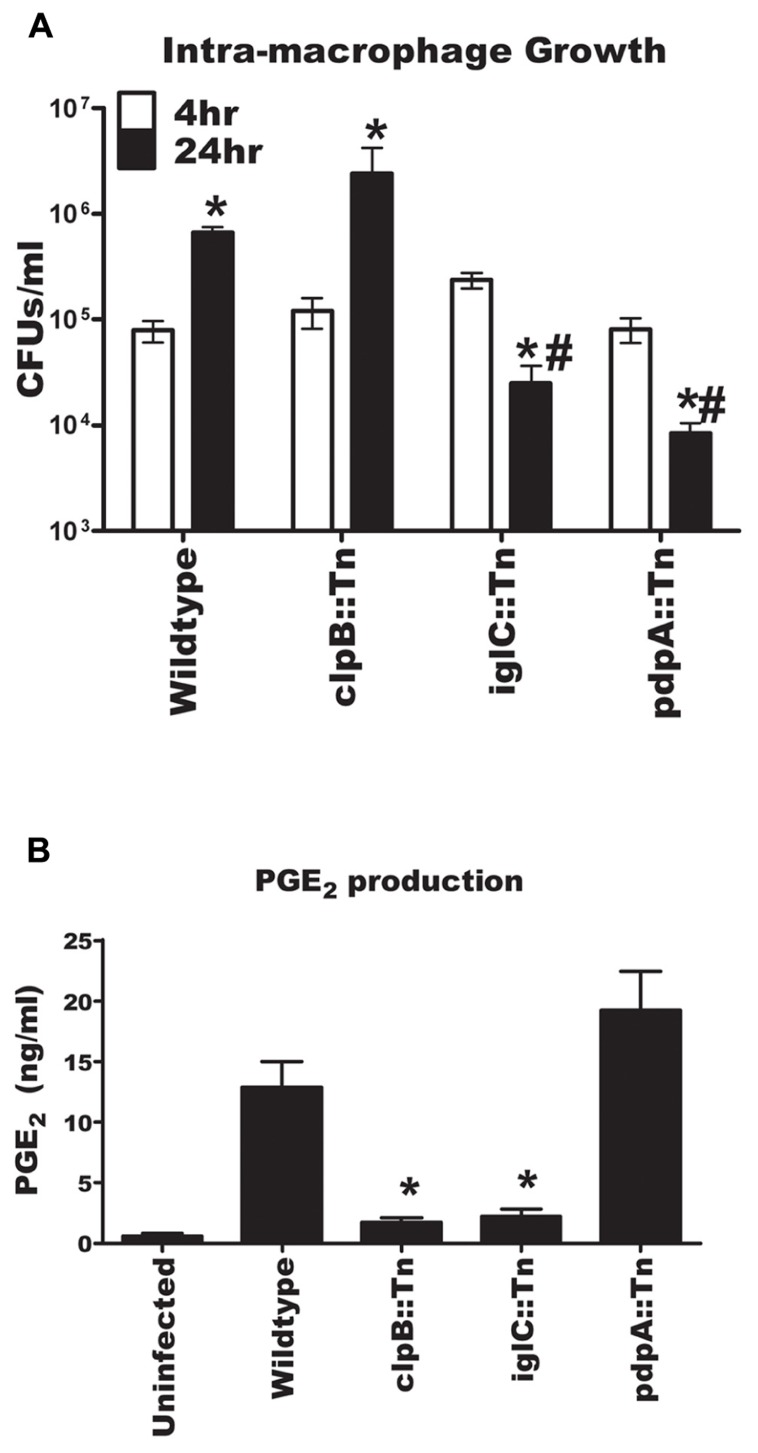
**Dissociation of intracellular growth and the induction of PGE_2_ from bone marrow-derived macrophages (BMDMs)**. **(A)** BMDMs were inoculated with U112, U112 *clpB*::Tn, U112 *iglC*::Tn, or U112 *pdpA*::Tn at an MOI of 100:1. CFU were determined at 4 and 24 h post-inoculation. Data represents three independent experiments and expressed as the means ± SEM. Asterisk “*” denotes statistical difference (*p* ≤ 0.05) from corresponding 4 h sample. ^#^BMDM denotes statistical difference (*p* ≤ 0.05) from 24 h U112-infected BMDM (*n* = 3). **(B)** BMDMs were inoculated with U112, U112 *clpB*::Tn, U112 *iglC*::Tn, or U112 *pdpA*::Tn at an MOI of 100:1. Twenty-four hours after inoculation supernatants were collected and PGE_2_ concentration was determined. Data represents three independent experiments and expressed as the mean ± SEM. Asterisk “*” denotes statistical difference (*p* ≤ 0.05) from U112-infected BMDM.

To determine if intracellular growth was required for *F. novicida* induction of PGE_2_ synthesis we inoculated BMDM at an MOI of 100:1 with U112, U112 *clpB*::Tn, U112 *pdpA*::Tn, and U112 *iglC*::Tn and counted intracellular CFUs over time. We used an MOI 100:1 to maximize differences in intracellular CFUs at 4 and 24 h post-inoculation. At higher MOIs extensive cell death of BMDMs by 24 h post-inoculation made it difficult to measure intracellular growth (data not shown). The number of intracellular bacteria was determined at 4 and 24 h post-inoculation, while the concentration of PGE_2_ in supernatants was determined at 24 h post-inoculation. The U112 *clpB*::Tn strain grew within BMDM similarly to wild-type U112, while the U112 *pdpA*::Tn and U112 *iglC*::Tn strains failed to grow in BMDM (**Figure 5**). In fact, there were fewer intra-macrophage U112 *pdpA*::Tn and U112 *iglC*::Tn bacteria at 24 h post-inoculation than at 4 h post-inoculation. Wild-type U112 and U112 *pdpA*::Tn were able to induce PGE_2_ synthesis, while U112 *clpB*::Tn and U112 *iglC*::Tn did not. The fact that *pdpA*::Tn induced PGE_2_ synthesis without intra-macrophage growth and *clpB*::Tn did not induce PGE_2_ synthesis while still able to grow in the macrophage demonstrates dissociation between intracellular growth and the ability of *F. novicida* to induce infected BMDM to synthesize PGE_2_.

## DISCUSSION

The induction of PGE_2_ synthesis by LVS-infected macrophages disrupts T cell responses allowing LVS to persist in the host (Woolard et al., 2007, 2008). We demonstrate here that induction of PGE_2_ synthesis by infected BMDMs is conserved among *F. novicida*, *F. holarctica*, and *F. tularensis*. Synthesis of PGE_2_ by U112-infected macrophages allowed us to screen the comprehensive U112 two allele transposon mutant library to identify *Francisella* genes that are potentially involved in the induction of PGE_2_ synthesis by *Francisella*-infected macrophages. Our screen identified 20 genes that when disrupted resulted in strains that failed to induce PGE_2_ synthesis by *F. novicida*-infected BMDM. These 20 genes are highly conserved in all sequenced *Francisella* subspecies (Charity et al., 2007, 2009; Nano and Schmerk, 2007; Meibom et al., 2008). Eighteen of the genes identified in this study either mapped to the FPI or represent positive transcriptional regulators of the FPI (Nano and Schmerk, 2007). Seventeen of the 20 identified genes have been demonstrated to be involved in mouse virulence (Su et al., 2007; Weiss et al., 2007). Most, but not all of these genes, encode proteins that have been implicated in escape from the phagosome and intracellular growth (Su et al., 2007; Weiss et al., 2007). The data presented here suggest these gene products may be responsible for the induction of PGE_2_ biosynthesis in infected BMDM independent of their role in phagosomal escape and intracellular growth.

The FPI likely encodes a secretion system. The FPI proteins PdpB, VgrG, DotU, IglA, and IglB are homologous to T6SS proteins from other bacterial pathogens (Ludu et al., 2008; de Bruin et al., 2011; Broms et al., 2012; Robb et al., 2012). The FPI was initially identified in *F. novicida* via mutations in *iglA* and *iglC* that resulted in *F. novicida* strains that no longer replicated within macrophages (Gray et al., 2002). Recent work has identified the FPI genes that encode proteins required for intracellular growth and include *pdpA*,* pdpB*,* dotU*, *vgrG*, *iglABCDEFHJ*, and potentially *iglG* and *iglI* (Nano et al., 2004; Santic et al., 2005, 2007; de Bruin et al., 2007, 2011; Bonquist et al., 2008; Broms et al., 2011). The genes *pdpC*, *pdpD*, *pdpE*, and *anmK* are not required for intracellular growth (de Bruin et al., 2011). Our screen demonstrated that disruptions in FPI genes *dotU*, *vgrG*, *pdpBC*, and *iglABCGEDFGHIJ* resulted in U112 strains unable to induce PGE_2_ synthesis by infected macrophages. At this time we are unsure whether all gene products are necessary, or whether some mutants where identified due to polar effects of transposon insertions. This is possible as the FPI is believed to be organized in two operons (Nano and Schmerk, 2007). Future work will be necessary to define which FPI gene products are truly necessary for induction of PGE_2_ synthesis from infected macrophages. Disruptions in *pdpADE* and *anmK* did not impair the bacteria’s ability to induce synthesis of PGE_2_. We were not surprised that *pdpD* and *anmK* mutants are not impaired, as we believe the mechanism of induction of PGE_2_ synthesis is conserved between *Francisella* strains. The *anmK* gene is not present in LVS while the *pdpD* is truncated in LVS and presumably non-functional (Ludu et al., 2008). The deletion of *pdpE* from *F. novicida* had no effect on the bacteria’s ability to grow in macrophages or cause disease. At this time the role of PdpE in FPI function is unknown (de Bruin et al., 2011). PdpA is involved in both intracellular growth and virulence. However, PdpA is not believed to be a component of the FPI secretion system (Schmerk et al., 2009a,b) which may explain why the *pdpA::Tn* mutant is still capable of inducing PGE_2_ synthesis. Some of the transposon mutants (*pdpE*::TN, *pdpD*::Tn, and *anmK*::Tn) were capable of inducing enhanced PGE_2_ secretion from infected macrophages. The mechanism behind this is unknown and future work will be done to examine this phenomenon. Regardless, it is clear that disruption of *F. novicida*’s genes in the FPI diminishes its ability to induce PGE_2_ synthesis from infected macrophages.

There are six genes located outside of the FPI that when disrupted resulted in strains unable to induce the synthesis of PGE_2_ from U112 infected macrophages. Four of those (*trmE*, *sspA*, *mglA*, and *mglB*) have previously been identified to encode positive transcriptional regulators of genes found both in the FPI and outside the FPI (Baron and Nano, 1998; Lauriano et al., 2004; Charity et al., 2007, 2009; Schmerk et al., 2009a,b). The work of the Dove laboratory has clearly identified other transcriptional regulators which include CaiC, CphA, PigR, and SpoT in *F. tularensis* LVS (Charity et al., 2009). The two allele mutant library lacks transposon insertional mutants in *spoT* and *pigR*, while the *caiC* and the *cphA* transposon mutant strain induced PGE_2_ synthesis from macrophages. This result suggests differential transcriptional regulation of the FPI between U112 and LVS; however future work would be required to corroborate this observation. RpoB is a component of the RNAP catalytic core responsible for the transcription of genes (Allison et al., 1985). The U112 *rpoB*::Tn was likely identified due to a general disruption of transcription. The fact the *rpoB*::Tn mutant failed to induce PGE_2_ synthesis would predict finding other components of the RNAP catalytic core. However, the two allele library did not contain mutants with transposon insertions in either *rpoA* or *rpoD*, while *rpoC*::Tn and *rpoZ*::Tn mutant strains induced PGE_2_ synthesis. ClpB, a stress response protein, has been previously demonstrated to be important in *Francisella* disease pathogenesis. A U112 *clpB* mutant was identified due to a delay in intra-macrophage growth, while a disruption of *clpB* in *F. holarctica* LVS resulted in a strain that could grow *in vitro* in macrophages, but failed to effectively multiply in mice (Gray et al., 2002; Meibom et al., 2008). We did not observe a intra-macrophage growth defect of the two allele *clpB*::Tn. In *Listeria monocytogenes* and *Porphyromonas gingivalis*, ClpB homologs are necessary for virulence during animal infections (Chastanet et al., 2004; Yuan et al., 2007). ClpB/ClpV homologs have been identified in other T6SS where their AAA+ ATPase activity supply energy for the protein secretion process (Mougous et al., 2006; Shalom et al., 2007; Shrivastava and Mande, 2008; Bonemann et al., 2009). ClpB regulates the protein levels of DnaK, FTL_0525, FTL_0311, FTL_0588, and FTL_0207 (Meibom et al., 2008). Since none of these genes were identified as necessary for induction of PGE_2_ synthesis, it would suggest ClpB may have other unidentified functions. It has not been demonstrated to regulate the protein levels of the FPI. Future work will be needed to define the mechanism of ClpB-mediated induction of PGE_2_ synthesis from infected macrophages, and whether this is through regulation of FPI genes, function of FPI gene products, or through a FPI-independent mechanism.

Infection of macrophages with U112, LVS, or Schu S4 results in the induction of PGE_2_. This demonstrates that the ability to induce PGE_2_ synthesis from infected macrophages is conserved among *F. tularensis* subspecies. In fact, U112 and Schu S4 induce more PGE_2_ than LVS at similar doses. This difference in PGE_2_ induction may be partially responsible for difference in virulence in these different subspecies. While we have previously demonstrated differences in innate immune responses to Schu S4, LVS, and U112 in intranasally inoculated mice, it is unknown if these different responses are due to differences in PGE_2_ induction (Hall et al., 2008). Further work will address this difference in PGE_2_ induction and the potential effect of PGE_2_ on disease pathogenesis. The demonstration that inactivation of FPI genes in *F. novicida* results in the inability to induce PGE_2_ biosynthesis and the fact that the FPI is highly conserved among all subspecies of *F. tularensis* would suggest that the mechanism of PGE_2_ induction is conserved among these subspecies. The fact that LVS *mglA*, *sspA*, and *dotU* mutant strains did not induce PGE_2_ synthesis further suggests the likelihood that *F. tularensis* subspecies *tularensis*, *F. tularensis* subspecies *holarctica*, and *F. tularensis* subspecies *novicida* have conserved mechanisms of induction of PGE_2_ synthesis. However, we cannot discount the possibility that *F. tularensis* subspecies *tularensis* may possess additional mechanisms for induction of PGE_2_ synthesis that *F. novicida* or *F. holarctica* do not.

The FPI is necessary for the organism to escape the phagosome and replicate in the cytosol (Nano et al., 2004; Santic et al., 2005, 2007; de Bruin et al., 2011). The reason the transposon insertion mutants we identified in U112 failed to induce PGE_2_ synthesis may be due to their failure to escape the phagosome and subsequently replicate. Failure to escape the phagosome may create a physiologic barrier between *F. tularensis* and the eukaryotic molecule that is responsible for sensing and responding to *F. tularensis.* There are many intracellular receptors that can recognize bacterial products (Franchi et al., 2009). ASC, a component of the inflammasome, and AIM2 (which recognizes *F. tularensis* DNA) are crucial for control of *Francisella* intra-macrophage growth *in vitro* and infection *in vivo* (Mariathasan et al., 2005; Fernandes-Alnemri et al., 2010; Jones et al., 2010). Inflammasome activation is also capable of inducing eicosanoid production (von Moltke et al., 2012). However, we believe that failure to escape into the cytosol is not the reason the transposon insertion mutant strains we identified in this study failed to induce PGE_2_ synthesis by infected macrophages. In other studies, *pdpA*::Tn and ∆*pdpA* *F. novicida* mutants fail to fully escape the phagosome (Mariathasan et al., 2005; Schmerk et al., 2009a,b). The U112 *pdpA*::Tn strain in the U112 two allele mutant library does not escape the phagosome to the same level as wild-type U112. Recently, 92 transposon mutant strains from the two allele mutant library were identified that did not escape the phagosome (Asare and Abu Kwaik, 2010). We showed all of these strains were able to induce PGE_2_. Thus, it is unlikely that the mutants we did identify failed to induce PGE_2_ solely because they failed to escape from the phagosome. Future work that identifies both the *F. tularensis* effector molecule and the corresponding eukaryotic binding partner will allow us to more definitively dissociate *F. tularensis* trafficking and induction of PGE_2_ synthesis from *Francisella*-infected macrophages.

Previous studies have identified 201 genes outside the FPI that are required for *Francisella* intra-macrophage growth (Qin and Mann, 2006; Maier et al., 2007; Asare and Abu Kwaik, 2010; Asare et al., 2010). U112 strains with insertions in any one of these 201 genes were all capable of inducing PGE_2_ synthesis from infected macrophages. We did not identify known *F. tularensis* auxotrophs as being defective in the ability to induce PGE_2_. Transposon insertions in *purA*, *purF*, *carA*, *carB*, and *pyrB* produce strains that have a defect in intracellular growth yet are able to induce macrophage synthesis of PGE_2_ (Maier et al., 2007; Quarry et al., 2007; Schulert et al., 2009). Our studies using U112, U112 *clpB*::Tn, U112 *pdpA*::Tn, and U112 *iglC*::Tn strains demonstrate dissociation between intra-macrophage growth, the ability of *F. tularensis* to fully escape the phagosome, and the ability to induce PGE_2_. These data also confirm our earlier report that UV inactivation of LVS, which inhibits replication, did not impact LVS’s ability to induce PGE_2_ synthesis from infected macrophages (Woolard et al., 2007). Further characterization and understanding of the molecular interactions between *F. tularensis* and eukaryotic cells that lead to the induction of PGE_2_ will provide new insight into tularemia pathogenesis.

## AUTHOR CONTRIBUTIONS

Matthew D. Woolard carried out all experiments except confocal and TEM microscopy. Lydia M. Barrigan and Adam S. Buntzman designed and carried out all confocal and TEM experiments. James R. Fuller and Joshua Bryan designed and generated strains used in study. Matthew D. Woolard drafted the manuscript. Colin Manoil aided in the design and use of the transposon library. Matthew D. Woolard, Thomas H. Kawula, Jeffrey A. Frelinger, and Colin Manoil designed and coordinated experiments and analyzed data. All authors read and approved the final manuscript.

## Conflict of Interest Statement

James R. Fuller is currently employed by Laboratory Corporations of America. All work conducted by James R. Fuller was prior to his employment to Laboratory Corporations of America, as such LaboratoryCorporations of America have no proprietary claim to any work presented in this publication.

## SUPPLEMENTARY MATERIAL

The Supplementary Material for this article can be found online at http://www.frontiersin.org/Microbial Immunology/10.3389/fmicb.2013.00016/abstract
